# Divergent activities of osteogenic BMP2, and tenogenic BMP12 and BMP13 independent of receptor binding affinities

**DOI:** 10.3109/08977194.2011.593178

**Published:** 2011-06-27

**Authors:** Stephen P Berasi, Usha Varadarajan, Joanne Archambault, Michael Cain, Tatyana A Souza, Abe Abouzeid, Jian Li, Christopher T Brown, Andrew J Dorner, Howard J Seeherman, Scott A Jelinsky

**Affiliations:** 1 Tissue Repair, Pfizer Research, 200 Cambridge Park Drive, Cambridge, MA 02140, USA; 2 Biological Technologies, Pfizer Research, 200 Cambridge Park Drive, Cambridge, MA 02140, USA

**Keywords:** Bone morphogenetic proteins, thrombospondin 4, tendon markers

## Abstract

Ectopic expression of recombinant human bone morphogenetic protein 2 (rhBMP2) induces osteogenesis, while ectopic expression of rhBMP12 and rhBMP13 induces the formation of tendon-like tissue. Despite their different *in vivo* activities, all three ligands bound to the type I bone morphogenic protein receptors (BMPRs), activin receptor-like kinase (ALK)-3 and ALK6, and to the type II BMPRs, activin receptor type-2A, activin receptor type-2B, and BMPR2, with similar affinities. Treatment of C3H10T1/2 cells with rhBMP2 activated SMAD signaling and induced expression of osteoblast markers including osteocalcin mRNA *(Ocn)*. In contrast, treatment with rhBMP12 or rhBMP13 resulted in a dose-dependent induction of a tendon-specific gene *(Thbs4)* expression with no detectable activation of SMAD 1, 5, and 8. Differential regulation of *Thbs4* and *Ocn* has potential utility as an *in vitro* biomarker for induction of tenogenic signaling. Such an assay also permits the ability to distinguish between the activities of different BMPs and may prove useful in studies on the molecular mechanisms of BMP tenogenic activity.

## Introduction

Bone morphogenic proteins (BMPs) are members of the transforming growth factor beta superfamily, which is a group of growth factors known to induce growth and differentiation of various cell types (Reddi 1998). Originally identified as components of de-mineralized bone that can induce bone and cartilage formation *in vitro* and *in vivo*, the BMP family now includes more than 30 related members. On the basis of sequence similarity, BMPs have been grouped into several subtypes. BMP2, 4, 6, and 7 are members of a major BMP family subtype that are responsible for inducing bone and cartilage formation (Wozney et al. 1988). Ectopic expression of BMP2 in rat muscle, for example, initially induces mesenchymal stem cell migration, proliferation, and chondrocytic differentiation. Chondrocytes subsequently calcify and are then replaced by mature bone. Studies on the functions of rhBMP2 and rhBMP7 in various preclinical models demonstrated increases in bone growth and regeneration (Cook and Rueger 1996; Rosen and Wozney 2002).

A distinct subset of related and highly conserved BMPs (BMP 12, BMP 13, and growth/differentiation factor 5 (GDF5)) is characterized by a lack of propensity to induce bone formation. Instead, molecules of this class induce the formation of cartilage and tendon-like structures *in vivo* (Chang et al. 1994; Wolfman et al. 1997). Administration of these BMPs after tendon injury in animal models results in increased rates of tendon repair and increased strength and stiffness of injured tendons (Aspenberg and Forslund 1999; Lou et al. 2001; Bolt et al. 2007; Seeherman et al. 2008). GDF5 has been evaluated as an inducer of spine fusion in an animal model (Jahng et al. 2004; Walsh et al. 2004) and as a treatment for degenerative disc disease in the clinic. More recently, polymorphisms in *Gdf5* were found to be associated with increased risk of tendinopathy (Posthumus et al. 2010).

BMP 12, BMP 13, and GDF5 share 82% amino acid sequence identity and similar functional properties (Wolfman et al. 1997). These molecules are also similar in sequence to osteogenic BMP family members (e.g. BMP12 and BMP2 share 55% amino acid sequence identity) and it has been suggested that BMP 12, BMP 13, and GDF5 can bind and signal through the same pathways as BMP2 and BMP4 (Mazerbourg et al. 2005). Both subclasses of BMPs initiate intracellular signaling through interaction with the type I receptors, activin receptor-like kinase (ALK)-3 and ALK6, and with the type II receptors, BMP receptor (BMPR)-2 and activin receptor 2B (ACVR2B; Mazerbourg et al. 2005). Specificity of response may in part be contributed to differential receptor ligand binding as ligand/receptor bindings has been found to be different among the BMP family members. BMPs bind their receptors as heterotetrameric complexes containing two type I and two type II receptors. Previous work has shown that BMP2 and BMP4 have higher binding affinity for the type I receptors, ALK3 and ALK6, and have lower binding affinity for the type II receptors, BMPR2, ACVR2A, and ACVR2B. BMP6 and BMP7 on the other hand can bind to the same receptors as BMP2 and BMP4, but bind the type II receptors with higher affinity than binding to the type I receptors. In contrast, GDF5 has been shown to preferentially bind ALK6 compared with ALK3 after forming a heteromeric complex with BMPR2 or ACVR2B (Nishitoh et al. 1996; Erlacher et al. 1998; Nickel et al. 2009). Activation of the ligand-receptor complex leads to signaling through the canonical SMAD pathway, but signaling can also proceed through the mitogen-activated protein (MAP) kinase pathway and possibly other pathways (Nohe et al. 2004).

Despite the high level of amino acid sequence identity and the strong *in vivo* phenotypes, the mechanism of action for BMPs which leads to the formation of tendon-like tissue vs. bone tissue is not well understood. In part, this is due to the lack of a specific and reproducible *in vitro* assay for measuring BMP tenogenic activity. The expression of two genes in particular, thrombospondin 4 (*Thbs4*) and tenomodulin (*Tnmd*), appears to be highly localized to tendon/ligament tissues, thereby making these genes potential tendon tissue-specific markers (Jelinsky et al. 2010). In contrast, biochemical markers of bone remodeling and growth have been well established and have been used for many years as useful tools to monitor bone health. Commonly, the detection of osteocalcin (*Ocn*), runt-related transcription factor 2 (*Runx2*), and osterix (*Sp7*) as markers has been used to determine early stages of bone formation. Additionally, alkaline phosphatase (ALP) activity and SMAD intracellular signaling have been monitored to quantify bone formation and BMP activity. In this study, we compare receptor binding affinities of tenogenic and osteogenic BMPs and show differential canonical BMPs signaling and activation of a novel tenogenic marker. These studies show that even though tenogenic (BMP 12 and BMP 13) and osteogenic (BMP2) BMPs bind the same receptors with high affinity they signal much differently and result in differential activation of osteogenic and tenogenic markers.

## Methods

### 

#### Ethics statement

Protocols for the use of animals in this work were approved by the Wyeth Animal Care and Use Committee, and were in accordance with the National Institute of Health's standards established by the Guidelines for the Care and Use of Experimental Animals.

#### Reagents and proteins

Mature forms of rhBMP12 and rhBMP13 were expressed in *Escherichia coli* in inclusion bodies. Inclusion bodies were solubilized in 8.0 M Urea, 100 mM DTT, 20 mM Tris, pH 8.4, and the pH was adjusted to 6.5. The unfolded protein was captured on an SP-Sepharose column equilibrated with 25 mM HEPES, 25 mM MES, 8.0 M Urea, and pH 6.5, and eluted with a linear 0–1.0 M NaCl gradient over 10 column volumes. Refolding was achieved by rapid dilution of the protein into refolding buffer (50 mM Tris, 5.0 mM EDTA, 1.0 M NaCl, 2% CHAPS, 0.03% Reduced Glutathione, 0.03% Oxidized Glutathione, pH 8.4). After 7 days, the refolded protein was purified using the SP Sepharose column, as described above, followed by size-exclusion chromatography on two 21.5 × 60-cm TOSOH G3000SWL columns in tandem in 6.0 M Urea, 300 mM NaCl, and 3% acetic acid. rhBMP2 was produced and purified from CHO cells as previously described (Wang et al. 1990). For initial quality control comparisons, some rhBMP12 was purified from CHO cells. The purified ligands were analyzed by reduced (with β-mercaptoethanol) and nonreduced SDS-PAGE analysis and Coomassie blue staining. Additionally ligand purity was determined by analytical HPLC gel filtration (data not shown). Proper refolding was determined by circular dichroism (CD) spectroscopy using mammalian expressed protein references. The spectra were taken at 0.1 mg/ml in 50 mM acetic acid, using a Jasco J-810 CD spectropolarimeter (Jasco, Easton, MD, USA) and a 1-mm quartz sample cell equilibrated at 20°C. Scans between 190 and 260 nm were performed at a scan rate of 50 nm per min.

#### Ectopic assays

Adult, male, Sprague-Dawley rats were obtained from Charles River Laboratory (Wilmington, MA, USA) and maintained on a 12-h:12-h light:dark cycle with food and water *ad libitum*. Rats were anesthetized using isoflorane (1–3% by inhalation). A small incision of approximately 0.5 cm in length was made on the medial aspect of the hamstring using a No. 15 scalpel blade. A small pocket was made in the intramuscular space with the blunt end of the scalpel to receive the implant. Implants consisted of 25 µg of rhBMP12, rhBMP13, or rhBMP2 lyophilized onto rat demineralized bone matrix particles. Implants were placed into the intramuscular space and sutured into position with 4-0 Vicryl cutting material. The surrounding soft tissue and skin were closed using absorbable suture material and skin staples.

#### Histology

Tissue sections (5-6 µm thick) were stained with Weigert's Safranin O stain (Polyscientific, Bay Shore, NY, USA) for histological analysis. Images were semi-quantitatively scored by a three-point scale based on density and fibroblast morphology. Multiple fields within an image were examined by three different blinded reviewers, and the final score was an average of all fields from all reviewers. A score of one was given to images containing primarily loosely packed fibroblast having a thin, spindle-shaped morphology, along with low proteoglycan and extracellular matrix content. A score of three was given for images showing densely packed fibroblasts with large cytoplasms, high fibrotic extracellular matrix levels, and high proteoglycan levels. Images with an “intermediate” number of fibroblasts were given a score of two. Prior to histological evaluation, ectopic implants were removed from animals, and a faxitron microradiograph apparatus (Faxitron X-ray Corp., Wheeling, IL, USA) was then used for the visualization of *in vivo* bone formation.

#### Cell culture conditions

The murine mesenchymal stem cell line C3H10T1/2 (clone 8) was purchased from ATCC (CCL-226; Manassas, VA, USA). Low passage cells were cultured in 1X Minimum Essential Media (MEM; Invitrogen, Carlsbad, CA, USA) supplemented with 10% heat inactivated fetal bovine serum (FBS) without the addition of antibiotics. Exponentially growing cells (2 × 10^5^ cells at a density of 2 × 10^5^/ml) from passages 3 to 6 were cultured in clear flat bottom 12-well plates overnight. The following day, the media was replaced with MEM supplemented with 1% FBS without antibiotics. A single dose of BMP was added to the culture, and the cells were incubated at 378C in 5% CO_2_.

#### BMP activity assays

C3H10T1/2 cells were transiently transfected in 12-well plates using Fugene-6 (Roche, Basel, Switzerland) with a BMP-responsive firefly luciferase reporter (BRE-luc; Korchynskyi and ten Dijke 2002) vector, and a pRL-TK renilla luciferase transfection efficiency control vector. After 24 h, cells were treated with either 1, 10, or 100nM of rhBMP12, rhBMP13, or rhBMP2 in Dulbecco's Modified Eagle Medium supplemented with 1 % FBS or with BMP free control medium, and assayed for either luciferase activity (after 24 h) or ALP activity (after 4 days). Luciferase activity was determined after cell lysis using the Dual-Glo Luciferase Assay System (Promega, Madison, WI, USA). All experiments were performed in triplicate. In all cases, firefly luciferase levels were normalized to the corresponding renilla luciferase levels in a given well, yielding final values in relative light units. ALP activity assays were performed as described previously (Thies et al. 1992).

#### Western blotting

Equal amounts of protein (80 µg/lane) or supernatants were concentrated (80% concentrated) using Microcon YM-10 (Millipore, Billerica, MA, USA) spin columns and underwent SDS-PAGE under reducing conditions using NuPage MOPS 4–12% gels (Invitrogen). Gels were transferred to nitrocellulose and probed with polyclonal antibodies against phospho-SMAD 1/5/8 (Cell Signaling Technology, Beverly, MA, USA; 1:750), β-actin (Abcam, Cambridge, MA, USA), b-tubulin (Sigma-Aldrich, St. Louis, MO, USA), Thrombospondin-4 (R&D Systems, Minneapolis, MN, USA), or with the anti-HA.11 monoclonal antibody (Covance, Princeton, NJ, USA).

#### TaqMan real time quantitative PCR

RNA was isolated and purified from cells using RNeasy kit (Qiagen, Valencia, CA, USA) according to the manufacturer's instructions. Relative mRNA levels were measured by TaqMan real time quantitative PCR using Assay-on-Demand TaqMan reagents and primers (Applied Biosystems, Foster City, CA, USA). TaqMan was performed using an ABI PRISM 7900 Sequence Detection System (PE Applied Biosystems). Expression levels were determined by the ΔΔCT method, normalized to *Gusb* mRNA levels, and then presented as the ratio of expression of each target mRNA in BMP-treated samples compared with the expression of the same mRNA in the control sample.

#### Receptor binding

Fusion proteins consisting of the BMPR extracellular domain linked to the human IgG Fc region (ALK1-Fc, ALK2-Fc, ALK3-Fc, and ACVR2A-Fc) were either purchased (R&D Systems), or synthesized in-house (ALK6-Fc, ACVR2B-Fc, and BMPRII). Receptor Fc chimeras were immobilized on anti-human IgG Fc biosensor tips (ForteBio, Menlo Park, CA, USA). Binding assays using sixfold serial dilutions of human BMPs in solution were performed using Octet RED (ForteBio). Association time was set at 10min and dissociation time was set at 30min. Binding affinities were calculated using ForteBio Data Acquisition 6.3 software (ForteBio). Affinities were derived by fitting the kinetic data to a 1:1 Langmuir binding model as described previously (Heinecke et al. 2009) utilizing global fitting algorithms.

#### Generation and use of ALK3 kinase dead adenovirus

cDNAs encoding HA-tagged, dominant negative ALK3 (K261R; Gupta et al. 2000), or GFP were used to create adenoviral vectors in 293 cells. Large scale purification by CsCl gradient ultracentrifugation was done at Viraquest (North Liberty, IA, USA). C3H10T1/2 cells were infected with a multiplicity of infection of 250 and then incubated with adenoviruses for 6h at 378C in MEM supplemented with 1% FBS without antibiotics. Media were replenished, rhBMP12 or rhBMP13 (10 or 100nM) was added, and cells incubated for an additional 3 days.

## Results

### Purification ofrhBMP2, rhBMP12, andrhBMP13

**Figure 1:**
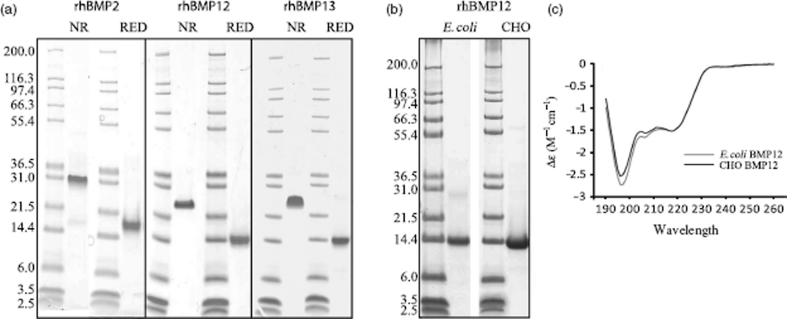
Characterization of purified rhBMP2, rhBMP12, and rhBMP13. (a) The purified proteins were collected and analyzed under reducing (RED) and nonreducing (NR) conditions using 12% polyacrylamide slab gels. (b) SDS-PAGE analysis of rhBMP12 purified from *E. coli* or CHO cells reduced with b-mercaptoethanol. (c) CD spectroscopy of BMP12 purified from *E. coli* or CHO cells.

The mature domain of rhBMP2 was purified from CHO cells overexpressing rhBMP2.The mature domains of rhBMP12 and rhBMP13 were purified from *E. coli* inclusion bodies and refolded. Small quantities of CHO expressed rhBMP 12 was purified and used as reference material to validate refolded *E. coli* expressed proteins. Purified proteins (5 mg) were characterized by SDS-PAGE under reducing conditions (with β-mercaptoethanol) or nonreducing conditions (Figure 1a). No glycosylations are predicted by the primary sequence of BMP 12 and both CHO-expressed and *E. coli* expressed rhBMP 12 had the same apparent molecular mass (approximately 14 KDa), on reduced SDS-PAGE gel consistent with the predicted molecular mass from the amino acid sequence of native BMP 12 without any post translational modifications (Figure 1b). The apparent size of the proteins determined by unreduced SDS-PAGE analysis is consistent with homodimer formation. The purity of the protein was determined by SDS-PAGE and HPLC (data not shown) and suggested that all three proteins were greater than 95% pure. CD spectroscopy suggested that *E. coli*-derived and CHO-derived rhBMP 12 have similar secondary structure confirming that *E. coli*-derived protein is properly folded (Figure 1c).

### Distinct tissues mediated by ectopic expression ofrhBMP2, rhBMP12, and rhBMP13

**Figure 2:**
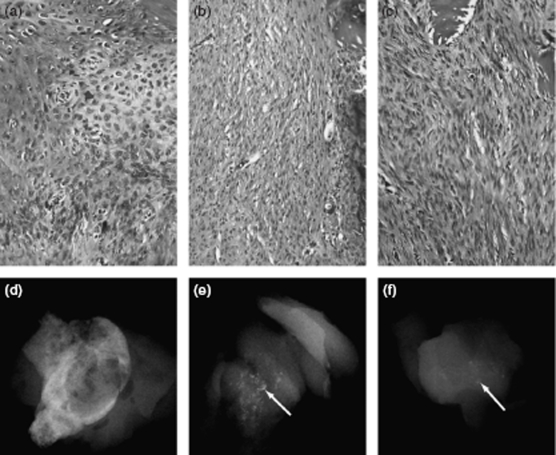
Histology and X-ray analysis of BMP-treated implants after 14 days. Saffranin O stain of representative sections of implants containing (a) 25 µg of rhBMP2, (b) 25 µg of rhBMP12, or (c) 25 µg of rhBMP13. Faxitron X-ray images of representative implants containing (d) 25 µg of rhBMP2, (e) 25 µg of rhBMP12, or (f) 25 µg of rhBMP13. The implants containing rhBMP2 consists of mostly bone, whereas the implants containing rhBMP12 or rhBMP13 consists of only small little bone spicules (arrows).

After 14 days of implantation, microscopic evaluation of ectopic implants containing rhBMP2, rhBMP 12, or rhBMP 13 showed increased fibroblast content and activity in a dose-dependent manner (Figure 2a–c, Table I). rhBMP2-containing implants induced the proliferation or migration of many more cells than either rhBMP12 or rhBMP13-containing ectopic implants. Fibroblasts in rhBMP12- and rhBMP 13-containing implants aligned in parallel to each other and displayed a wave-like pattern, which is characteristic of embryonic or neonatal tendon. In contrast, rhBMP2-containing implants contained endochondral bone that was encompassing small islands of hypertrophic cartilage. rhBMP2 formed ectopic bone in all implants, whereas only high doses of rhBMP 12 or rhBMP 13 in implants induced ectopic bone formation. However, the ectopic bone induced by rhBMP 12- and rhBMP 13-containing implants was visibly less than that induced by rhBMP2-containing implants. The ectopic bone formed by rhBMP2 encompassed most of the implant area, whereas only small spicules of ectopic bone were formed by rhBMP 12 and rhBMP 13 (Figure 2d–f, and Table I). Low doses of rhBMP2 induced low levels of ectopic bone and the accumulation of loosely packed quiescent fibroblasts. In rhBMP2 implants, the fibroblasts did not exhibit any observable parallel alignment as was observed in the rhBMP 12- and rhBMP 13-containing implants.

**Table I:** Ectopic cell assay

	Cellular activity*			Bone Formation†		
		
Dose (µg)	rhBMP2	rhBMP 12	rhBMP 13	rhBMP 2	rhBMP 12	rhBMP 13
25	3	2.7	2.7	6/6	3/6	1/6
5	3	2.3	2.2	6/6	1/6	2/6
1	2.7	1.5	2.2	4/6	1/6	0/6
0.2	1.5	1.2	2	3/6	0/6	0/6
0	1.2	1.2	1.2		0/6	

* Cellular activity based on number and morphology of fibroblasts in the ectopics (see Methods)

† Number of implants that contain evidence of bone formation.

All implants formed a hard callus and were easily isolated from surrounding tissues. RNA expression analysis identified many tendon marker genes including *Col1a1, Col1a2, Eya1, Eya2, Scx, Epha4, Tnmd, and Thbs4* that exhibited changes in relative level in rhBMP 12- and rhBMP 13-induced ectopic tissues (Table II). In contrast, many bone marker genes including *Bglap* (osteocalcin) *Sp7* (osterix) and *Alpl* (ALP) exhibited changes in relative level in rhBMP2-induced ectopic tissues (Table II). Ectopic tissue induced by both rhBMP 12 and rhBMP2 increased expression of cartilage marker genes including *Sox9, Acan*, and *Col2a* (Table II). The increased expression of tendon-related genes was not specific to rhBMP12 treatment as most of these genes were also increased in the rhBMP2-treated ectopics. However, increases in bone-related genes appeared to be selective to rhBMP2-treated ectopics.

**Table II:** Fold change in RNA expression of musculoskeletal markers in rat ectopic tissue.

Name	Description	rhBMP12	rhBMP13	rhBMP2
Bone markers				
*Bglap2*	Bone gamma-carboxyglutamate 2	NS	NS	3588
*Sp7*	Osterix	2	1.7	86
*Alpl*	Alkaline phosphatase, tissue-nonspecific	7.2	3.9	129
*Runx2*	Runt-related transcription factor 2	NS	NS	4.2
Tendon markers				
*Scx*	Scleraxis	5.1	4.3	4.9
*Tnmd*	Tenomodulin	5.5	5.2	6.9
*Thbs4*	Thrombospondin 4	2.3	2.0	NS
Cartilage markers				
*Agc1*	Aggrecan 1	9.6	7.4	6.2
*Col2a1*	Collagen, type II, alpha 1	NS	NS	14.1
*Col11a1*	Procollagen, type XI, alpha 1	4.9	4.6	15.2
*Sox9*	Sex determining region Y-box 9	2.9	2.2	2.4

Note: NS, not significantly regulated, *p* > 0.05.

### rhBMP2, rhBMP12, and rhBMP13 bind the same receptors

To determine whether the observed differences in BMP-mediated gene expression were due to a difference in canonical BMPR–ligand binding, receptor-ligand binding affinities were calculated. Using Bio-Layer Interferometry (Octet) analysis, binding curves were generated from experiments in which immobilized receptor extracellular domain—IgG Fc fusion proteins were exposed to purified BMPs. rhBMP2, rhBMP12, and rhBMP 13 bound the same type I and type II BMPRs. The type I receptors ALK3 and ALK6 bound to rhBMP2, rhBMP 12, and rhBMP 13 with high affinity (*K*_D_) and bound all three BMPs with similar association (*k*_on_) and dissociation (*k*_off_) rates (Table III). While ALK2 did bind to rhBMP2, but not to rhBMP 12 or rhBMP 13, the affinity of this interaction was orders of magnitude weaker than the affinity of rhBMP2 for ALK3 or ALK6. rhBMP2, rhBMP 12, and rhBMP 13 also bound the type II receptors ACVR2A, ACVR2B, and BMPRII with comparable levels of affinity. Each of the three BMPs interacted with ACVR2A and BMPRII with a binding affinity (*K*_D_) that is more than 10-fold less than the affinity observed with the type I receptors. Contrastingly, ACVR2B exhibits affinity for rhBMP 12 and rhBMP 13 that is similar to that observed for type I receptors. Calculated binding affinities for receptor–ligand interactions in which all three BMP ligands bound tightly were very similar, thus distinguishing this state of interaction from weaker interactions, such as those observed with ACVR2A and BMPR2.

**Table III:** Binding affinities (kD, nM) of BMP ligands as determined by surface plasmon resonance

	Type I receptor							Type II receptor					
		
	ALK1	ALK2		ALK3		ALK6		ACVR2A		ACVR2B		BMPR2	
							
	Mean	Mean	SD	Mean	SD	Mean	SD	Mean	SD	Mean	SD	Mean	SD
rhBMP2													
*k*_on_ × 10^−4^ (M^−1^ s^−1^)	NB	24	0.80	608	68	428	89	355	329	764	25	716.3	85.0
*k*_off_ × 10^3^ (s^−1^)	NB	54.8	0.8	6.8	0.8	6.4	0.7	146.6	53.5	61.1	1.8	191.0	22.1
*K*_D_ (kin) (nM)	NB	227		1.1	0.0	1.6	0.5	60.0	40.5	8.0	0.5	26.7	0.1
rhBMP12													
*k*_on_ × 10^−4^ (M^−1^ s^−1^)	NB	NB		507	155	648	69	1334	84	1293	159	926	3
*k*_off_ × 10^3^ (s^−1^)	NB	NB		16.4	1.4	4.0	0.8	228	17.7	13.8	6.0	322	27.7
*K*_D_ (kin) (nM)	NB	NB		3.4	0.8	0.6	0.1	17.1	2.4	1.0	0.3	52.1	3.2
rhBMP13													
*k*_on_ × 10^−4^ (M^−1^ s^−1^)	NB	NB		497	178	769	47	605	412	259	25	570	22
*k*_off_ × 10^3^ (s^−1^)	NB	NB		8.4	3.1	3.8	1.3	79.8	28.0	4.1	1.4	160	8.1
*K*_D_ (kin) (nM)	NB	NB		1.7	0.0	0.5	0.1	15.1	5.7	1.6	0.4	28.1	2.5

Notes: The data were fitted to a kinetic model (1:1 Langmuir binding) from which *K*_D_ (kin) is calculated as *k*_off_ (× 10^3^ s^−1^)/*k*_on_ (× 10^−4^M^−1^ s^−1^). NB, no binding above background detected; ND, not determined.

### Distinct activation of bone, cartilage, and tendon gene markers with rhBMP2, rhBMP12, and rhBMP13 in C3H10T1/2

**Figure 3:**
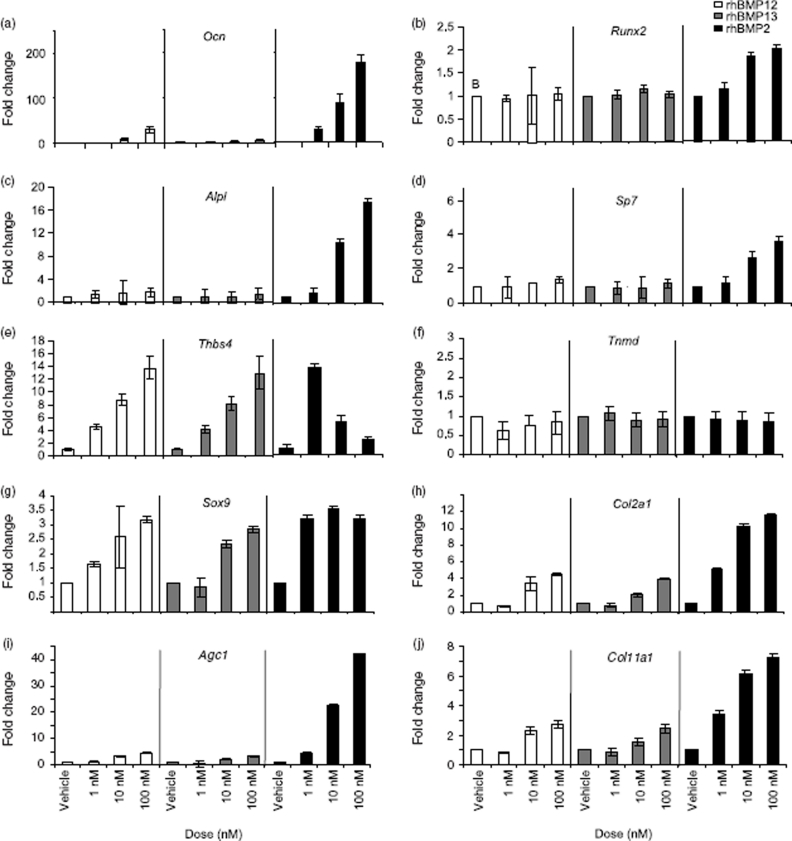
Expression of bone, tendon, and cartilage markers by BMPs in C3H10T1/2 cells. mRNA levels were determined by Taqman for the bone markers (a) *Ocn* (osteocalcin), (b) *Runx2* (runt-related transcription factor 2, (c) *Alpl* (ALP), and (d) *Sp7* (osterix), the tendon markers (e) *Thbs4* (thrombospondin 4) and (f) *Tnmd*, (tenomodulin) and the cartilage markers (g) *Sox9* (SRY-box containing gene 9), (h) *Col2a1* (collagen, type II, alpha 1), (i) *Acan* (Aggrecan 1) and (j) *Col11a1* (collagen, type 11, alpha 1).

Analysis of ectopic tissues is complicated by the multiple distinct cell types present including skeletal muscle, inflammatory cells, and fibroblasts. To begin to distinguish the reproducible differential activity of rhBMP2, rhBMP12, and rhBMP13, activation of musculoskeletal marker genes was investigated in the easily cultured BMP-responsive mesenchymal cell line, C3H10T1/2. C3H10T1/2 cells cultured with rhBMP2 but not rhBMP12 or rhBMP13 produced high levels of the bone marker mRNAs *Ocn, Runx2, Alpl*, and *Sp7*. Cells treated with relatively high concentrations of rhBMP12 or rhBMP13 (100nM) made relatively low levels of *Ocn* mRNA and did not express detectable levels of the other bone markers examined (Figure 3a–d). Each of the three BMPs tested had the effect of increasing levels of the collagen marker mRNAs *Sox9* and *Col2a1*, but rhBMP2 treatment increased *Col2a1* by approximately 2.5-fold over that observed with rhBMP12 or rhBMP13 treatment. Furthermore, rhBMP2 treatment increased *Sox9* mRNA levels at much lower doses than did rhBMP12 and rhBMP13 (Figure 3).

*Thbs4* and *Tnmd* were previously identified as tendon-specific markers in rat and human tissues (Jelinsky et al. 2010). mRNA and protein analyses support the tissue specificity of *Thbs4* in mouse tendon (Supplemental Figure 1). A 3-day treatment of C3H10T1/2 with rhBMP12 or rhBMP13 increased *Thbs4* mRNA levels in a dose-dependent manner up to 15-fold (Figure 3e). Treatment of C3H10T1/2 cells with low concentrations of rhBMP2 (1 and 5nM) resulted in increased *Thbs4* mRNA levels, but unlike rhBMP12 and rhBMP13, higher concentrations of rhBMP2 failed to increase *Thbs4* mRNA levels further (Figure 3e). Neither of the BMPs appeared to affect *Tnmd* mRNA levels, and *Scx* was not expressed under the conditions tested.

### Distinct intracellular pathway activation with rhBMP2, rhBMP12, and rhBMP13

**Figure 4:**
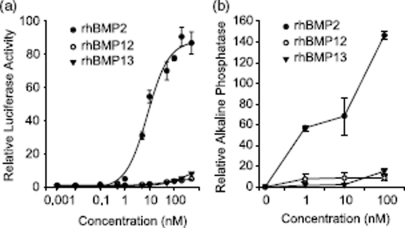
BMP activity in C3H10T1/2 cells. (a) C3H10T1/2 cells infected with BRE-Luc construct were treated with increasing concentration of rhBMP2, rhBMP12, or rhBMP13. (b) ALP activity in C3H10T1/2 cells following 2-day incubation with 0, 1, 10, or 100nM of rhBMP2, rhBMP12, or rhBMP13.

As C3H10T1/2 cells were responsive to all three BMPs and replicate many of the molecular changes observed *in vivo* after ectopic expression, we explored differences in intracellular signaling mechanisms in this cell line. BMP ligands differentially activate a SMAD 1, 5, 8 dependent BRE-luciferase reporter (BMP response element) in C3H10T1/2 cells. rhBMP2 treatment led to a more than 100-fold induction of BRE-luciferase reporter activity EC50, ∼8 nM) compared with control. rhBMP12 induced the lowest levels of BRE-luciferase reporter activity at 6-fold (EC50, ∼52 nM) that of control, whereas rhBMP13 induced only slightly more BRE-luciferase reporter activity at 10-fold (EC50, ∼272 nM; Figure 4a). In a similar trend, neither rhBMP12 (10 nM) nor rhBMP13 (10 nM) caused considerable increases in ALP activity, whereas rhBMP2 treatment (10 nM) did result in a clear increase in ALP activity (60-fold increase). At 100nM rhBMP2, there was a 14-fold higher increase in ALP activity compared with changes in ALP activity observed with rhBMP12 or rhBMP13 (Figure 4b).

**Figure 5:**
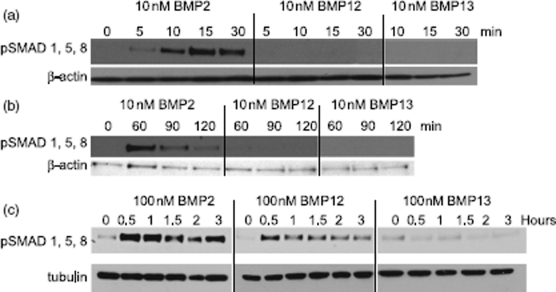
Induction of SMAD signaling by BMPs in C3H10T1/2 cells. Cell lysates from rhBMP2, rhBMP12, or rhBMP13 treated C3H10T1/2 cells were analyzed by western blot with antibodies to phosphorylated SMAD 1/5/8 and to β-actin or tubulin. Cells were treated with (a)10 nM BMPs for 0 to 30 min, (b) 10 nM BMPs for 0 to 120 min or (c) treated with 100 nM BMPs for 0 to 3 h.

rhBMP2 induced phosphorylation of SMADs 1, 5, and 8 much more potently than rhBMP12 or rhBMP13. C3H10T1/2 cells treated with 10 nM rhBMP2 exhibited induced SMAD 1, 5, and 8 phosphorylation 30min after treatment, but this decreased after 60 min (Figure 5a,b). In contrast, C3H10T1/2 cells treated with rhBMP12 (10 nM) or rhBMP13 (10 nM) did not exhibit any detectable changes in phosphorylated SMADs after up to 2 h of treatment (Figure 5b). At higher doses of rhBMP2 (100 nM), high levels of phosphorylated SMADs were maintained for at least 3h. Higher doses of rhBMP12 (100 nM) induced SMAD 1, 5, and 8 phosphorylation, but this effect was much less than that observed with rhBMP2 (Figure 5c).

### Kinase dead ALK3 expression blocks BMP12 induction of Thbs4

**Figure 6:**
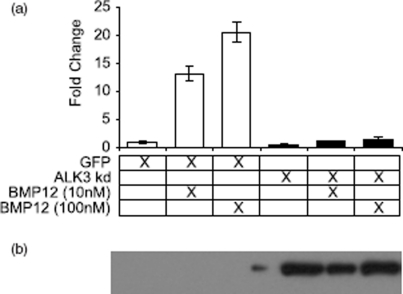
Kinase dead ALK3 blocks *Thbs4* induction. (a) mRNA levels for *Thbs4* were determined by Taqman after treatment with kinase dead ALK3 and rhBMP1 2. Error bars represent SD (*N* = 3). (b) anti-HA western of protein extract from the infected cells to detect expression of the kinase dead ALK3.

*Thbs4* is the only tendon enriched gene that showed differential activity between rhBMP12, 13, and 2 in C3H10T1/2 cells. Expression of a kinase-dead dominant-negative type I BMPR (ALK3) completely abrogated the increased *Thbs4* mRNA levels observed with rhBMP12 (Figure 6). Our data also indicate that rhBMP12 and 13 can cause increased *Thbs4* expression at concentrations that do not result in considerable increases in phosphorylated SMADs, but in a way that involves signaling through type I receptors. We have not yet examined other type I receptor possibilities because these cells are not known to express any other relevant receptor. On the basis of quantitative PCR analysis, C3H10T1/2 cells express high levels of *Alk1, Alk2, and Alk3*, but do not express detectable levels of *Alk6* (Supplemental Figure 2). As the BMP ligands tested in this study do not bind ALK1, and only BMP2 binds ALK2 with very low affinity (Table III), our efforts were focused on ALK3.

## Discussion

Despite high amino acid sequence identity among BMPs 2, 12, and 13, divergent *in vivo* activities are observed for these ligands. Ectopic expression of rhBMP2 results in endochondral bone growth, while ectopic expression of rhBMP12 or rhBMP13 induces the formation of aligned, collagen-secreting fibro-blasts. Here, we show that, despite very similar binding affinities for the same BMPRs, tenogenic and osteogenic BMPs signal through the canonical BMP SMAD pathway in different ways that result in greater levels of characteristic bone marker mRNAs, or increases in tendon marker *Thbs4* mRNA. These results demonstrate the utility of *Thbs4* as a specific *in vitro* tenogenic BMP marker that can be regulated by BMP 12 and BMP 13.

We have attempted to describe how BMP ligands bind the same canonical receptors but illicit distinct *in vivo* responses. Although our binding affinity data were only investigated biochemically, our results clearly identified differences in binding affinities between the ligands and the different receptors tested. These differences lead to hypothesis on how these ligands illicit their biological responses. It is however important to note that interaction with additional cellular receptors, cofactors, or inhibitors may alter the *in vivo* binding affinities.

It is possible that low levels of BMP activity are necessary to signal tendon differentiation and that high levels are required for bone formation. In support of this, treatment of cells with a relatively high dose (100 nM) of rhBMP12 or rhBMP13 can result in low *Ocn* mRNA levels and in the formation of endochon-dral bone, indicating that high levels of BMP 12 or BMP 13 might have an effect that is similar to comparably low levels of BMP2 (1 nM). Although bone and mRNA markers for bone are clearly detected following rhBMP12 or rhBMP13 treatment, the amount of actual bone and levels of markers for bone are relatively low compared with that observed upon rhBMP2 treatment. Moreover, ectopic implants treated with low doses of rhBMP2 do not resemble ectopic implants treated with rhBMP12 and rhBMP13 based on histological examination. Fibroblasts found in ectopic tissue treated with low doses of rhBMP2 do not display the characteristic wave-like pattern seen after ectopic expression of rhBMP12 or rhBMP13. It may be possible that the development of bone prevents the development of tendon tissue.

Expression of the tendon marker mRNA *Thbs4* is relatively high shortly after treatment with low concentrations of rhBMP2, whereas treatment with higher concentrations of this ligand results in no detectible *Thbs4* mRNA. As an interesting parallel, during muscle development, signaling through the BMP2 pathway can lead to decreased smooth muscle cell marker expression (Hayashi et al. 2006) and can block development of muscle from forelimb bud mesenchyme (Duprez et al. 1996; Amthor et al. 1998). BMP2 can also inhibit terminal differentiation of myoblasts and is capable of converting their differentiation pathway into the osteoblast lineage (Katagiri et al. 1994). Similarly, BMP12 and BMP 13 can block myogenic differentiation, but these ligands fail to initiate osteoblast differentiation (Inada et al. 1996).

Our receptor binding data correlate well with others' showing that BMP2/4 and BMP12/13/GDF5 have high affinity for the type I receptors and lower affinity for the type II receptors (Nickel et al. 2009). Specificity may in part be due to the nature of the interaction. BMP2, BMP4, and GDF5 initially bind type I receptors followed by binding to the lower affinity type II receptors while BMP6 and BMP7 have higher affinity for the type II receptors and bind type II receptors followed by binding to type I receptors (Nishitoh et al. 1996; Heinecke et al. 2009; Nickel et al. 2009).

Although rhBMP2 and rhBMP12/13 bind the same BMPRs, their biological effects are very different. BMP2 signals through the canonical SMAD pathway leading to bone formation, while BMP 12 and 13 induce expression of the tendon marker, *Thbs4*, at doses where there is little SMAD 1, 5, or 8 signaling. There are a number of possible explanations for why these ligands have different *in vitro* and *in vivo* signaling properties. Our data suggests that the tenogenic BMP 12 and BMP 13 have higher affinity for ALK6 compared with ALK3, while BMP2 appears to have similar affinities for ALK3 and ALK6. It is possible this slight affinity change could alter downstream SMAD signaling. It has also been suggested that slight changes in the orientation of the extracellular ligand/receptor complex formation are enough to change the conformation of the intracellular kinase domains to drastically affect downstream signals (Nickel et al. 2009). Additionally, BMP 12 and BMP 13 may interact with a different set of receptors/co-receptors that may be less than optimal for efficient downstream canonical SMAD signaling compared with that observed for BMP2, but signaling still may occur through a noncanonical pathway. BMPs are known to signal through MAP kinases and p38 in bone (Guicheux et al. 2003; Noth et al. 2003). We have not yet evaluated the potential role of this pathway, but it represents interesting potential future work. Furthermore, there may be additional unknown cofactors that could interact with ligand–receptor complexes in ways that impact canonical signaling and there may be crosstalk between canonical and noncanonical BMP signaling pathways that affects relative levels of osteogenic vs. tenogenic gene expression. Given that rhBMP2 is capable of binding ALK2 weakly, it is possible that BMP2 signaling through ALK2 results in sufficient differentiation to promote bone formation. Activating mutations in ALK2 increase SMAD phosphorylation and can lead to fibrodysplasia ossificans progressiva, a rare disabling disease characterized by heterotopic ossification (Fukuda et al. 2009; Kaplan et al. 2009). Finally, specificity maybe based on receptor expression patterns. While the exact cells that responded to BMP ligands are not known, six of the receptors (*Alk1, Alk2, Alk3, Acvr2, Acvr2b* and *Bmpr2*) are expressed in the three major musculoskeletal tissues likely to be the sight of action, bone, muscle, and tendon (Jelinsky et al. 2010). The expression of *Alk6* is below the sensitivity of the assay (Jelinsky et al. 2010). Assuming that the protein expression correlates to mRNA expression, this suggests that BMP2, BMP 12, and BMP 13 have the capacity to signaling through ALK3 in the target tissue of interest.

We also report on the characterization of a molecular marker for monitoring the activity of rhBMPs involved in neotendon/ligament formation. The assay relies on changes in tendon-selective *Thbs4* mRNA levels (Jelinsky et al. 2010). *Thbs4* mRNA levels are high in normal tendon cells, and this marker appears to be selective for tendon tissue. Therefore, *Thbs4* mRNA levels might be a good surrogate for the formation of tendon tissues. We optimized assay conditions to obtain reproducible effects on changes in *Thbs4* mRNA levels, and show that the dose-dependent increase in *Thbs4* mRNA is specific for BMPs that exhibit *in vivo* neotendon/ligament formation ability.

Additionally, we show that BMP-induced *Thbs4* is blocked by overexpression of a dominant negative BMP type I receptor ALK3. Although our results suggest that ALK3 is important, inhibiting BMP 12-induced *Thbs4* mRNA may be the result of a number of different mechanisms including blocked receptor activity or loss of receptor specificity/selectivity due to titration of ALK3 interacting proteins (for example type II receptors). While the exact mechanism is not known, it is clear that altering BMPR-mediated signaling affects the ability of BMP 12 to induce *Thbs4* mRNA expression.

Related members of the BMP family are capable of affecting *Thbs4* mRNA levels, but in different ways. rhBMP2 causes an increase in *Thbs4* levels only at low ligand concentrations, while a dose-dependent increase in *Thbs4* mRNA levels is observed with rhBMP12. As treatment with rhBMP2 showed a bell-shaped response for the induction of *Thbs4* but not for other endpoints, one might expect that a larger dose range of rhBMP 12 might show a similar response for induction of *Thbs4*. However, BMP 12 is relatively insoluble and higher concentrations than that reported here result in an insoluble protein fraction which is predicted to decrease activity on all end points.

We also note that under our culture conditions, these cells are not differentiating into tendon cells and little change in cell morphology is observed. Other tendon-associated markers including *Scx* (scleraxis), *Col1a1, Six1*, and *Six2* are not induced under these conditions (data not shown). *Thbs4*, as a marker that is highly and selectively expressed in tendon cells, is essential for dissecting the different functions of various BMP ligands. In particular, for the tenogenic BMPs, *Thbs4* mRNA levels provide a more sensitive way to assess BMP activity compared with the BRE-luciferase reporter assay, which may be limited to detecting canonical BMP signaling through SMAD activation. Our work compared the characterization of *in vivo* ectopic gene expression and cultured cell lines. We note that many parallels exist in genes expressed in both systems but many differences exist. We have used a cell culture system to begin to elucidate distinct mechanism of activity between the different BMP molecules and to use an easily cultured cell line to accurately measure activity. More work is needed to determine if these differences exist *in vivo* as well as to determine the exact cell type that responds to BMPs *in vivo*.

Most established *in vitro* BMP assays monitor bone related formation activity, such as increases in ALP activity and *Ocn* mRNA levels. While the tenogenic BMPs studied in this work impact levels of ALP activity and *Ocn* mRNA levels, their activity is considerably reduced compared with true osteogenic BMP family members such as BMP2. Rat osteosarcoma cell ALP activity increases by 50% following treatment with BMP 12 (Furuya et al. 1999). However, in C3H10T1/2 cells, the increases in ALP activity resulting from rhBMP12 and rhBMP13 treatments are 100-fold less than the activity induced by rhBMP2, and this is consistent with previous work in C3H10T1/2 and C2C12 cells using recombinant adenovirus (Luu et al. 2007). Measuring changes in *Thbs4* mRNA levels represents a much more sensitive and specific way to measure tenogenic activity from BMPs compared with other established BMP-related assays. This assay has the potential to be used to supplement traditional methods in screening for tenogenic activity and in evaluating specific functions of different BMPs. The utility of *Thbs4* mRNA levels in the study of tendon development may be equal to, or greater than, the utility of *Ocn* in studies of bone development due to the tissue specificity and sensitivity that we describe.

## Supplementary Material

Supplementary Figure 1. Tissue specificity of *Tnbs4* in mouse tendon as shown by mRNA and protein analyses.

Supporting Figure 2. Gene expression in C3H10T1/2 cells.
