# Supplementary figures and images for: Divergent activities of osteogenic BMP2, and tenogenic BMP12 and BMP13 independent of receptor binding affinities
Source: Growth Factors. 2011 Jun 27;29(4):128–39. doi: 10.3109/08977194.2011.593178 (PMC3154542; doi:10.3109/08977194.2011.593178)

10T 1/2

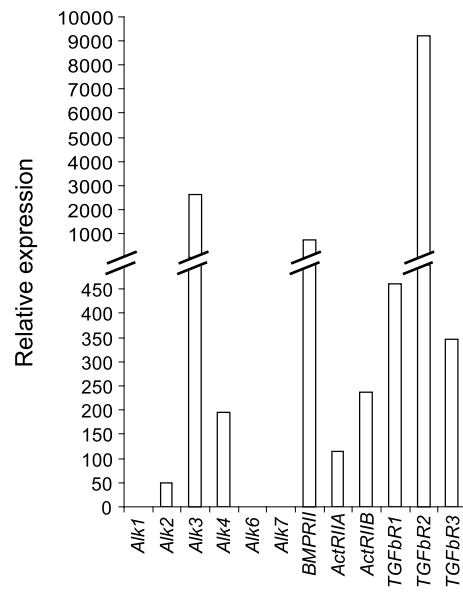

Supplement: Supporting Figure 2. Gene expression in C3H10T1/2 cells. [file SD2.pdf]
