# Supplementary material for: Divergent activities of osteogenic BMP2, and tenogenic BMP12 and BMP13 independent of receptor binding affinities
Source: Growth Factors. 2011 Jun 27;29(4):128–39. doi: 10.3109/08977194.2011.593178 (PMC3154542; doi:10.3109/08977194.2011.593178)
Supplement: Supplementary Figure 1. Tissue specificity of Tnbs4 in mouse tendon as shown by mRNA and protein analyses. [file SD1.pdf]

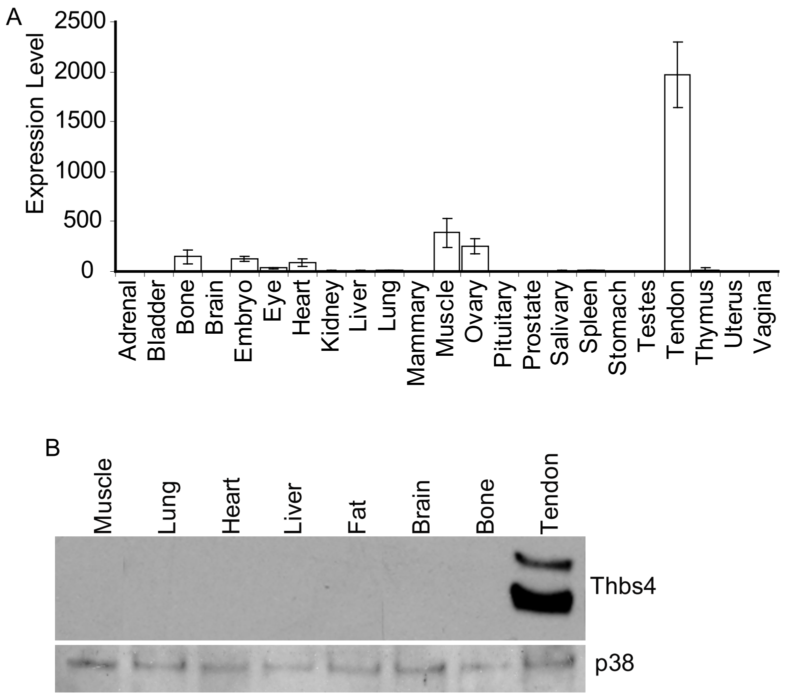
A
Expression Level
2500
2000
1500
1000
500
0
Adrenal
Bladder
Bone
Brain
Embryo
Eye
Heart
Kidney
Liver
Lung
Mammary
Muscle
Ovary
Pituitary
Prostate
Salivary
Spleen
Stomach
Testes
Tendon
Thymus
Uterus
Vagina
B
Muscle
Lung
Heart
Liver
Fat
Brain
Bone
Tendon
Thbs4
p38
